# Shedding light on the shadows: oxidative stress and its pivotal role in prostate cancer progression

**DOI:** 10.3389/fonc.2024.1393078

**Published:** 2024-05-07

**Authors:** Marek Biesiadecki, Mateusz Mołoń, Krzysztof Balawender, Zofia Kobylińska, Sabina Galiniak

**Affiliations:** ^1^Institute of Medical Sciences, Rzeszów University, Rzeszów, Poland; ^2^Institute of Biology, Rzeszów University, Rzeszów, Poland

**Keywords:** oxidative stress, nitrosative stress, oxidative damage of protein, prostate cancer, cancer progression

## Abstract

**Objectives:**

Data on oxidative protein damage, total antioxidant capacity (TAC) and lipid peroxidation in progression of prostate cancer remain elusive. So far, the influence of the presence of perineural invasion on the level of oxidative stress has not been described. Additionally, there is limited data on the level of oxidative stress in patients’ urine.

**Methods:**

We compared the levels of oxidative stress markers in serum and urine in 50 patients with prostate cancer depending on the tumor stage and histological grade, the Gleason score, and the presence of perineural invasion.

**Results:**

We found a significantly de-creased level of serum thiol groups and TAC in participants with prostate cancer. Similarly, serum Amadori products and malondialdehyde (MDA) were higher in patients than in healthy men. There was a significantly decrease in TAC and a significantly increased MDA in the urine of prostate cancer patients. As the stage of cancer increased, a decrease in the thiol group concentration and TAC as well as an increase in the concentration of lipid peroxidation products in the serum was observed. The serum level of advanced oxidation protein products (AOPP) increased in the group with Gleason scores greater than 7. Furthermore, serum thiol groups and TAC were reduced in the group with Gleason >7 as compared to Gleason <7. The presence of perineural invasion significantly reduced serum and urinary TAC and increased urinary AOPP concentration.

**Conclusions:**

These results indicate a significant role for oxidative damage in prostate carcinogenesis and its progression. Characterizing oxidative and nitrosative damage to proteins may be useful in designing targeted therapies for prostate cancer patients.

## 1 Introduction

Prostate cancer is one of the most frequently diagnosed cancers in developed countries and the fifth leading cause of death, with an estimated 1.4 million diagnoses worldwide in 2020 (1, 2). The incidence and mortality rates of prostate cancer are closely related to age, with the highest incidence seen in elderly men (3). Adenocarcinoma is the most common histological type of prostate tumors. Approximately 95% of prostate cancers develop in the acini of prostatic ducts (4). The etiology and risk factors for prostate malignancy are not well defined. However, factors such as age, race/ethnicity, genetic predisposition, family history, metabolic syndrome, obesity, environmental influences, and lifestyle are often associated with its development (5, 6). Oxidative stress, caused by a redox imbalance between pro-oxidants and antioxidants, contributes to carcinogenesis and cancer progression, including urogenital cancers (7–9). Increased production of reactive oxygen species (ROS) or impaired antioxidant defenses raise intracellular ROS levels, causing oxidative damage to various cellular components such as nucleic acids, proteins, and lipids, and activating intracellular proto-oncogenic signaling (10). Cancer cells often maintain high levels of oxidative stress, using ROS to modulate signaling pathways for growth, proliferation, and evasion of apoptosis (11). The relationship between oxidative stress and the development of prostate cancer has been frequently discussed (12, 13). However, data on oxidative protein damage, antioxidant capacity, and lipid peroxidation in subjects at high risk for prostate cancer remain elusive. Moreover, the associations between prostate cancer progression and oxidative stress is not fully understood. Prevention, early detection, and more effective treatment strategies for prostate cancer are essential, given its significance as a public health problem, particularly in Western countries with an ageing population (3, 14). Therefore, it seems crucial to identify markers that can be easily and cheaply determined in serum and/or urine and correlate with the stage and grade of prostate cancer.

This study aimed to investigate the changes and differences in oxidative stress parameters in the blood, serum, and urine of patients with prostate cancer compared to a control group. We also sought to find an association between the levels of the estimated markers and the stages or grades of prostate cancer. To the best of the authors’ knowledge, this study represents the first to examine the impact of perineural invasion on oxidative stress levels in patients with prostate cancer.

## 2 Materials and methods

### 2.1 Ethical issues

The study protocol was accepted by the Bioethics Committee of Rzeszow University with authorization numbers 2022/037 and 2022/090. The study was conducted in strict adherence to the ethical standards laid out in the World Medical Association Declaration of Helsinki regarding human subject research. Informed consent was obtained from each study participants.

### 2.2 Participant selection

The single-center, cross-sectional study included 50 men with prostate cancer, ranging in age from 55 to 86 years. These individuals had no history of medication or treatment for their condition prior to the study. Participants were recruited from the Clinical Department of Urology and Urology Oncology at the Municipal Hospital in Rzeszow, Poland, between March and September 2022. Comprehensive data on demographics, disease history, and familial cancer history were meticulously collected.

Inclusion criteria: Patients qualified for radical laparoscopic prostatectomy based on ultrasound-guided transrectal biopsies (utilizing ≥10 biopsy cores). All specimens were assessed by an experienced urogenital pathologist. Reports were issued following ISUP/World Health Organization (WHO) guidelines. The classification of the study group was based on the Union for International Cancer Control (UICC) 8th edition (2017), using the Tumor, Node, Metastasis (TNM) classification for prostate cancer staging (15). Grading was based on the Gleason score, with the 2019 International Society of Urological Pathology grade classification employed (16).]. An additional histopathological report parameter utilized in the study was perineural invasion.

Exclusion criteria: Refusal to participate in the study, incomplete medical documentation, prior radio- or chemotherapy, smoking, chronic alcohol use, history of endocrine disorders, other malignancies, diabetes mellitus, dyslipidemia, obesity (BMI >30), and infectious or inflammatory diseases.

The control group consisted of 45 healthy men, age-matched with the study group, who were selected among healthy volunteers. These participants had no history of cancer or chronic diseases and had not taken any drugs, including vitamins and supplements, for at least one month prior to the study. All volunteers in the control group had no lower urinary tract symptoms, normal PSA levels, and had normal digital rectal examinations. Socioeconomic status and dietary habits, as assessed during medical interviews, were similar across all participants.

### 2.3 Materials

All chemicals were purchased from Sigma-Aldrich (Poznan, Poland). The enzyme-linked immunosorbent assay kits for 3-nitrotyrosine and 4-hydroxy-nonenal were procured from Immunodiagnostic AG (K7829, Bensheim, Germany) and Wuhan Fine Biotech Co., Ltd. (EU0187, Wuhan, China), respectively. Absorptiometric analysis was conducted using a Tecan Infinite 200 PRO multimode reader (Tecan Group Ltd.; Männedorf, Switzerland). Measurements were made in triplicate, unless indicated otherwise. Results were standardized to protein or creatinine concentrations as appropriate, unless specified otherwise.

### 2.4 Sample collection

Peripheral blood (5 mL) was drawn after fast overnight using a Sarstedt S-Monovette system, followed by centrifugation and serum storage at −80°C for subsequent analyses. Urine samples were collected into 50 mL sterile containers from the initial morning void, centrifuged, and the supernatants stored under the same conditions.

### 2.5 Hematological and biochemical analysis

Blood counts were determined using an automated standard hematology analyzer (Siemens Healthineers, Germany). Serum glucose, creatinine, and urea were analyzed using standard laboratory methods (Cobas c501, Roche Diagnostics, Mannheim, Germany). Coagulological determinations were estimated on an ACL TOP 300 CTS Coagulation Analyzer (Instrumentation Laboratory, Werfen Headquarters, Barcelona, Spain). The international normalized ratio (INR) was assayed using the RecombiPlasTin 2G kit from Instrumentation Laboratory and the activated partial thromboplastin time (APTT) was estimated using the APTT-SP kit, also from Instrumentation Laboratory. The direct potentiometric measurement of potassium in blood serum was measured with a liquid ion-exchange electrode.

### 2.6 Biochemical procedures

#### 2.6.1 Protein assay

The protein concentration was determined using the method of Lowry et al. (17). Briefly, 250 μL of the Lowry reagent was applied to a 96-well plate. Then 50 μL of diluted serum was applied to each well and incubated at room temperature for 10 min. Subsequently, 25 μL of the Folin-Ciocalteu reagent was added and incubated for 30 minutes before measuring absorbance at 750 nm.

#### 2.6.2 Creatinine assay

Creatinine in urine was measured using Jaffé method (18).

#### 2.6.3 AOPP

Advanced oxidation protein products (AOPP) were determined by the method of Witko-Sarsat et al. (19). 200 μL of diluted serum and urine were added to wells of a 96-well plate and 20 μL of acetic acid was added to each well. Absorbance was measured at 340 nm against a blank. AOPP concentration is expressed in nmol chloramine-T equivalents/mg protein.

#### 2.6.4 3-Nitrotyrosine assay

The 3-nitrotyrosine concentration in serum was assessed with the 3-nitrotyrosine ELISA kit (Immundiagnostik AG), according to the manufacturer’s protocol. The results were expressed in nmol/mg protein.

#### 2.6.5 Thiol group assay

The level of thiol groups was determined by the method proposed by Ellman (20). 20 μL of serum and 2 μL of 5,5′-dithiobis-(2-nitrobenzoic acid) (DTNB; 10 mg/mL of 0.1 M phosphate buffer, pH 8.0) were added to 100 μL 0.1 M phosphate buffer, pH 8.0 to wells of a 96-well plate. The samples were incubated in the dark at 37°C for 1 h and the absorbance was measured at 412 nm against a blank. The thiol group content was calculated on the basis of a standard curve using glutathione as a standard and expressed in mmol/L.

#### 2.6.6 Characterization of Amadori product by the NBT assay

The content of the Amadori product was estimated using the method of Johnson et al. (21). 100 μL of serum or diluted urine were added to wells of a 96-well plate, followed by 100 μL of the nitro blue tetrazolium reagent in 0.1 M carbonate buffer, pH 10.35 and the plate was incubated at 37°C for 2 h. The absorbance was measured at a wavelength of 525 nm. The Amadori products were estimated using an extinction coefficient of 12,640 M-1 cm-1 for monoformazan (22). Measurements were made in duplicate.

#### 2.6.7 Total antioxidant capacity (TAC) measured by method with ABTS^•^

ABTS^•^ (2, 2’-azino-bis (3-ethylbenzothiazoline-6-sulphonic acid)) was formed by the reaction of 7 mM of ABTS solution with 2.45 mM of potassium persulfate, incubated for 24 hours at room temperature and protected from light. Briefly, appropriate amounts of serum samples or urine were added to a ABTS^•^ solution, diluted such that 200 µl of the solution had an absorbance of 1.0 ± 0.04 in a well. The absorbance reading was done at 734 nm after 6 minutes of reaction. The results were expressed in Trolox equivalents (μmol TE/L) (23).

#### 2.6.8 TAC measured by method with FRAP

TAC was determined colorimetrically by measuring the ferric reducing capacity of samples with 0.3 M acetate buffer, pH=3.6, 0.01 M 2,4,6-tripyridyl-s-triazine in 0.04 M HCl and 0.02 M FeCl3*6H2O mixed in 10:1:1 and serum or urine samples. Absorbance was measured at 593 nm after 20 minutes incubation. The results were expressed in Trolox equivalents (μmol TE/L) (24).

#### 2.6.9 MDA

Samples of serum or urine were mixed with 200 μL of mixture (1:1) of 0.37% thiobarbituric acid (TBA) and 15% trichloroacetic acid in 0.25 M HCl to precipitate protein. The samples were incubated for 40 min at 100oC and pH 2-3. Then, samples were centrifuged and the absorption of the supernatants was measured at a wavelength of 535 nm. The majority of TBA-reactive substances is MDA; therefore, the concentration of MDA in samples was expressed in μmol. The results were calculated using an absorption coefficient for MDA of 1.56×105 M−1 cm−1 (25).

#### 2.6.10 4-Hydroxy-nonenal assay

The 4-hydroxy-nonenal concentration was assessed with the 4-hydroxy-nonenal ELISA kit (Wuhan Fine Biotech Co., Ltd), according to the manufacturer’s protocol. The results were expressed in pg/mL.

### 2.7 Statistical analysis

Data presentation includes median and interquartile ranges, with distribution normality verified by the Shapiro-Wilk test. The Kruskal-Wallis or Mann-Whitney U test was applied to ascertain statistical significance. Data analysis was facilitated by the STATISTICA software suite (version 13.3, 2017, StatSoft Inc., OK, USA).

## 3 Results

Fifty men with prostate cancer were recruited for this study. Meanwhile, 45 healthy men were enrolled in the control group. The basic characteristics and clinical laboratory values of the study participants are shown in **Tables 1** and **2**. The classification of the study group was based on the 8th edition of the UICC 8th edition (2017), using TNM classification for the staging of prostate cancer (15). Grading was based on the Gleason score, using the ISUP 2019 grade classification (16). There were no statistical differences in basic characteristics between the study groups. The clinical hematological results were similar between the prostate cancer group and healthy subjects. The study groups had similar clotting indices. There were no differences in serum creatinine, glucose, urea and potassium levels between patients and healthy controls.

**Table 1:** General characteristics of the patients.

		Healthy controls	Prostate cancer group	p
n		45	50	
Age (years)	median	65.5	66	0.733
	Q1 − Q3	60.25 − 73	62.75 − 71.25	
BMI (kg/m^2^)	median	27.71	26.42	0.571
	Q1 − Q3	24.68 − 29.37	25.29 − 28.65	
WBC (10^3^/µL)	median	6.9	7.85	0.603
	Q1 − Q3	6.1 − 8.1	5.68 − 9.28	
Prothrombin time (s)	median	12.05	11.08	0.168
	Q1 − Q3	11.58 − 13	11.35 − 12.3	
Prothrombin time (%)	median	94	98	0.342
	Q1 − Q3	83.5 − 101	90.5 − 104	
INR	median	1	1	0.502
	Q1 − Q3	1 − 1.1	1 − 1.1	
APTT (s)	median	29.02	28.09	0.670
	Q1 − Q3	27.3 − 31.38	27 − 30.25	
Creatinine (mg/dL)	median	0.93	0.90	0.739
	Q1 − Q3	0.83 − 1.07	0.79 − 1.07	
Glucose (mg/dL)	median	99	101	0.547
	Q1 − Q3	94.5 − 103	96.5 − 102	
Urea (mg/dL)	median	34	37.5	0.290
	Q1 − Q3	28 − 44	31 − 53.75	
K^+^ (mmol/L)	median	4.35	4.55	0.138
	Q1 − Q3	4.2 − 4.6	4.28 − 4.8	

**Table 2:** Clinical characteristics of the study group patients.

Clinical stage (TNM)	n (%)
T1	14 (28%)
T2	20 (40%)
T3	16 (32%)
Gleason score	
<7 (ISUP grade 2)	22 (44%)
7 (ISUP grade 2/3)	19 (38%)
>7 (ISUP grade 4/5)	9 (18%)
Clinical grade	
G1	24 (48%)
G2	26 (52%)
Perineural invasion	
present	16 (32%)
absent	34 (68%)

In our study, we observed a similar level of serum AOPP, one of the most frequently determined markers of protein oxidative modification in patients with prostate cancer, compared to the control group (**Table 3**). Furthermore, there were no differences in serum 3-nitrotyrosine level. However, we found a significantly lower level of thiol groups in the serum of participants with prostate cancer. Similarly, another protein modification Amadori products was also higher in the serum of patients than in healthy men. The TAC measured by the ABTS^•^ method was significantly lower in men with prostate cancer than in healthy controls. However, this result was not confirmed in the TAC method with FRAP. One of the markers of lipid peroxidation − MDA − was significantly increased in cancer patients compared to healthy people. On the other hand, we did not observe any differences in 4-HNE concentration between the study groups. For urinary oxidative stress markers, we observed that there was no difference in the concentrations of AOPP, Amadori products, and TAC measured by ABTS• between the study groups. However, there was a significantly decreased TAC determined by FRAP and a significantly increased MDA in prostate cancer patients (**Table 3**).

**Table 3:** Markers of oxidative stress in healthy controls and patients with prostate cancer.

		Healthy controls	Prostate cancer group	p
Serum	AOPP (nmol/mg protein)	213.83 (192.02 − 291.16)	239.55 (195.37 − 425.47)	0.346
	3-nitrotyrosine (nmol/mg protein)	0.116 (0.11 − 0.14)	0.119 (0.1 − 0.16)	0.748
	Thiol groups (mmol/L)	643.92 (557.17 − 753.72)	573.87 (485.81 − 699.9)	0.044
	Amadori products (nmol/mg protein)	1745.69 (1587.43 − 2176.05)	1987.22 (1826.28 − 2299.01)	0.031
	TAC (ABTS^•^, μmol TE/L)	285.27 (271.48 − 294.88)	210.15 (201.85 − 217.55)	<0.001
	TAC (FRAP, μmol TE/L)	217.55 (184.24 − 237.92)	193.13 (168.65 − 225.12)	0.133
	MDA (μmol)	3.27 (3.11 − 3.41)	3.56 (3.39 − 4.02)	<0.001
	4-HNE (pg/mL)	345.62 (238.01 − 508.88)	412.08 (301.17 − 487.3)	0.746
Urine	AOPP (μmol/mmol creatinine)	12.72 (9.96 − 17.11)	14.87 (9.03 − 19.98)	0.538
	Amadori products (mmol/mmol creatinine)	0.352 (0.28 − 0.48)	0.384 (0.31 − 0.54)	0.467
	TAC (ABTS^•^, μmol TE/L)	856.83 (720.44 − 963.18)	758.85 (573.7 − 1026.94)	0.194
	TAC (FRAP, μmol TE/L)	564.90 (448.23 − 625.55)	451.44 (297.01 − 638.48)	0.047
	MDA (μmol/mmol creatinine)	0.86 (0.71 − 1.01)	1.15 (0.96 − 1.34)	<0.001

**Table 4** shows the concentrations of oxidative stress markers in serum and urine in men with prostate cancer, depending on the TNM classification. There was no statistical difference in the concentration of AOPP, 3-nitrotyrosine, and Amadori products between the study groups. However, we noted a significant difference in the concentration of serum thiol groups (T1 vs. T3 p = 0.002 and T2 vs. T3 p = 0.008). Furthermore, serum TAC decreased with tumor progression. As the stage of the cancer increased, an increase in the concentration of lipid peroxidation products in the serum was observed. On the other hand, urinary oxidative stress markers, with the exception of MDA (T1 vs. T3 p = 0.002 and T2 vs. T3 p = 0.01), were at similar levels among the study groups.

**Table 4:** Markers of oxidative stress in patients divided in groups considering the cancer stage depending on the TNM classification.

		T1	T2	T3	p
Serum	AOPP (nmol/mg protein)	238.35	219.40	378.77	0.123
	3-nitrotyrosine (nmol/mg protein)	0.137	0.117	0.113	0.941
	Thiol groups (mmol/L)	699.59	564.23	436.08	0.011
	Amadori products (nmol/mg protein)	1865.81	1996.79	2064.07	0.719
	TAC (ABTS^•^, μmol TE/L)	220.32	214.61	198.77	<0.001
	TAC (FRAP, μmol TE/L)	223.62	195.48	160.32	<0.001
	MDA (μmol)	3.39	3.56	4.31	0.001
	4-HNE (pg/mL)	246.9	417.42	508.19	0.002
Urine	AOPP (μmol/mmol creatinine)	12.21	17.83	15.37	0.594
	Amadori products (mmol/mmol creatinine)	0.384	0.357	0.382	0.405
	TAC (ABTS^•^, μmol TE/L)	763.28	696.62	959.94	0.589
	TAC (FRAP, μmol TE/L)	462	377.24	635.39	0.464
	MDA (μmol/mmol creatinine)	0.829	1.146	1.191	0.026

**Table 5** shows patients divided into groups based on Gleason score. The level of serum AOPP was increased in the group with Gleason scores greater than 7. Moreover, thiol groups in serum were reduced in the group with Gleason >7 as compared to Gleason <7. Similarly, serum TAC was decreased in patients with Gleason >7 as compared to those with Gleason lower than 7. The concentration of 4-HNE was increased in the group with Gleason scores greater than 7 when compared to the other study group (<7 vs >7 p < 0.001 and =7 vs >7 p = 0.032). Levels of serum 3-nitrotyrosine, Amadori products, MDA, and urinary oxidative stress markers were similar in the studied groups.

**Table 5:** Markers of oxidative stress in patients divided in groups considering Gleason grading system.

		<7	7	>7	p
Serum	AOPP (nmol/mg protein)	238.35	208.52	528.53	0.02
	3-nitrotyrosine (nmol/mg protein)	0.115	0.117	0.125	0.678
	Thiol groups (mmol/L)	700.81	555.68	488.69	0.002
	Amadori products (nmol/mg protein)	1829.38	2202.23	1962.49	0.086
	TAC (ABTS^•^, μmol TE/L)	215.74	215.24	198.77	<0.001
	TAC (FRAP, μmol TE/L)	207.85	216.93	166.00	0.007
	MDA (μmol)	3.40	3.42	4.01	0.077
	4-HNE (pg/mL)	258.68	417.42	527.98	<0.001
Urine	AOPP (μmol/mmol creatinine)	12.65	14.02	19.98	0.245
	Amadori products (mmol/mmol creatinine)	0.433	0.356	0.392	0.405
	TAC (ABTS^•^, μmol TE/L)	942.11	607.90	727.74	0.314
	TAC (FRAP, μmol TE/L)	634.48	446.00	498.74	0.70
	MDA (μmol/mmol creatinine)	0.853	1.174	1.306	0.054

Subsequently, we examined the level of biomarkers depending on the histological grade (**Table 6**). The serum levels of the markers tested, with the exception of TAC and MDA, were similar in all groups. The serum TAC was significantly decreased in the G2 group compared to patients with G1. Likewise, the MDA concentration was significantly higher in participants in G2 than in G1. Similarly, urinary TAC measured by FRAP was significantly lower in G2 compared to G1 patients. Furthermore, we observed increased MDA concentrations in G2 compared to G1 men.

**Table 6:** Markers of oxidative stress in patients divided in groups considering the cancer histological grade.

		G1	G2	p
Serum	AOPP (nmol/mg protein)	221.08	295.95	0.191
	3-nitrotyrosine (nmol/mg protein)	0.124	0.113	0.623
	Thiol groups (mmol/L)	623.49	555.31	0.37
	Amadori products (nmol/mg protein)	1982.92	2021.04	0.696
	TAC (ABTS^•^, μmol TE/L)	212.51	205.67	0.004
	TAC (FRAP, μmol TE/L)	220.27	166.01	<0.001
	MDA (μmol)	3.39	4.03	<0.001
	4-HNE (pg/mL)	390.8	474.68	0.124
Urine	AOPP (μmol/mmol creatinine)	14.43	19.97	0.999
	Amadori products (mmol/mmol creatinine)	0.321	0.382	0.049
	TAC (ABTS^•^, μmol TE/L)	728.12	912.93	0.731
	TAC (FRAP, μmol TE/L)	320.11	631.82	0.023
	MDA (μmol/mmol creatinine)	0.97	1.34	0.005

Finally, we investigated whether the presence of perineural invasion in patients with prostate cancer affects the level of oxidative stress. **Table 7** shows the level of biomarkers in patients with perineural invasion compared to participants without perineural invasion. The presence of perineural invasion significantly reduced serum and urinary TAC measured by FRAP and increased urinary AOPP concentration. No other differences were noted between men with and without perineural invasion.

**Table 7:** Markers of oxidative stress in patients divided in groups considering the presence of perineural invasion.

		Absent of perineural invasion	Presence of perineural invasion	p
Serum	AOPP (nmol/mg protein)	219.36	277.59	0.346
	3-nitrotyrosine (nmol/mg protein)	0.114	0.134	0.603
	Thiol groups (mmol/L)	542.56	586.98	0.223
	Amadori products (nmol/mg protein)	1962.49	2092.05	0.19
	TAC (ABTS^•^, μmol TE/L)	215.63	208.95	0.154
	TAC (FRAP, μmol TE/L)	207.92	176.32	0.036
	MDA (μmol)	3.52	3.84	0.147
	4-HNE (pg/mL)	326.92	453.66	0.102
Urine	AOPP (μmol/mmol creatinine)	10.63	22.56	<0.001
	Amadori products (mmol/mmol creatinine)	0.356	0.388	0.89
	TAC (ABTS^•^, μmol TE/L)	998.71	694.79	0.063
	TAC (FRAP, μmol TE/L)	638.48	296.42	<0.001
	MDA (μmol/mmol creatinine)	1.11	1.19	0.452

## 4 Discussion

Oxidative stress is a process in which the body is exposed to an excessive amount of reactive oxygen species (ROS) and reactive nitrogen species (RNS). These substances can damage cells and DNA, leading to various diseases, including cancer. There is evidence of a link between oxidative stress and the development of prostate cancer, although the mechanisms of this relationship are not fully understood (12, 26). Additionally, there is limited data on the level of oxidative stress in patients’ urine. The association of oxidative stress with prostate cancer progression also remains unclear.

In our study, there were no significant differences in serum and urine AOPP concentration for monitoring oxidative stress between healthy controls and prostate cancer patients. On the other hand, a recent study reported that AOPP concentrations were higher in prostate tissue from cancer patients than from healthy individuals (3.21 vs 2.42 μmol/mg, *p <*0.05) (27). Moreover, a significantly higher concentration in serum AOPP level was observed among prostate cancer patients than among healthy controls in a study by Koike et al. (28). The concentration of carbonyl groups, another marker of protein oxidative damage, increased among prostate cancer patients compared to healthy controls (1.09 ± 0.07 vs 2.3 ± 0.66 nmol/mg protein, *p* < 0.01) (29).

In addition to oxidative stress markers, we determined the concentration of 3-nitrotyrosine, which is a nitrosative stress marker. Some RNS interact with tyrosine residues, which may trigger the formation of 3-nitrotyrosine (30). In our study, we did not observe any differences in 3-nitrotyrosine concentration in the serum of cancer and healthy study participants. To our knowledge, the 3-nitrotyrosine concentration have not previously been measured in serum among patients with prostate cancer. Patients with prostate cancer were characterized by lower thiol groups in serum, consistent with previous studies (28, 31–33).

Reducing sugars can react non-enzymatically with the amino groups of proteins to form reversible Schiff bases, and then Amadori products (34). We determined significantly higher concentrations of Amadori products in serum from patients with prostate cancer than from healthy controls. However, the concentration of these products was at a similar level in the urine in both study groups. Amadori products undergo further complex reactions to form irreversibly cross-linked, heterogeneous fluorescent derivatives, termed advanced glycation end products (AGEs). Plasma carboxymethyllysine (CML), a major end-stage AGE, was significantly higher in prostate cancer cases than in controls (182 vs 152 μg/mL, *p* < 0.05) (34).

AGEs may react with intracellular proteins, altering their function or structure, resulting in increased free radical production directly or by binding to AGE receptors (RAGE) (35). Overexpression of RAGE and the RAGE polymorphism was suggested to be associated with prostate cancer development and poor prognosis (36).

Furthermore, we observed reduced serum (determined using ABTS) and urine (determined using the FRAP method) in men with prostate cancer compared to healthy controls. On the other hand, the total antioxidant parameter that captures radicals from erythrocytes and plasma was at a similar level in healthy controls and men with prostate cancer (28). Likewise, serum total antioxidant status was significantly lower in patients with prostate cancer compared to healthy individuals (1.396 ± 0.08 vs 1.815 ± 0.069 mmol/L, *p* < 0.001) (37).

We found increased concentrations of MDA in serum and urine from prostate cancer patients. Similarly, Veljković et al. reported that MDA concentration was increased in prostate tissue in people with cancer compared to healthy people (7.15 vs 4.42 nmol/mg, *p* < 0.001) (27). Elevated MDA concentrations were also determined in the circulation of patients with prostate cancer compared to healthy controls, which is consistent with our results (38, 39). Therefore, MDA may play an important role in the etiopathogenesis of prostate cancer (40).

We did not notice any difference in the 4-HNE concentration in the serum of individuals with prostate cancer and healthy individuals. A recent study revealed the absence of 4-HNE-protein adducts in prostate carcinoma tissue but increased 4-HNE-protein levels in the plasma of these patients (41). No difference was observed in the concentration of lipid hydroperoxide, one of the derivatives of lipid peroxidation, in plasma and erythrocytes from prostate cancer patients compared to controls (28). Moreover, urine F2-isoprostanes were not associated with prostate cancer incidence in the study by Yang et al. (34). On the other hand, an increase in F2-isoprostane concentration was observed in prostate patients in the study by Brys et al. (42).

In our study, as tumor stage increased, a decrease in the concentration of thiol groups and TAC was observed in serum, an increase in the concentration of MDA in serum and urine, and an increase in 4-HNE in serum. Similarly, with increasing tumor histological grade, we found a decrease in serum and urine TAC and an increase in MDA levels. Moreover, we did not observe any difference in the concentration of Amadori products in serum and urine in the study groups depending on the stage and grade of cancer. Subsequently, we checked the levels of oxidative stress markers in patients depending on the Gleason score. We found an increase in serum levels of AOPP and 4-HNE and a decrease in thiol group levels, as well as TAC in patients with Gleason greater than 7. One of the AGEs, CML, levels were lower in areas of the tumor, for both the epithelium and the surrounding stroma, compared to benign, but did not change significantly with the tumor grade group (43).

The Gleason score had no effect on plasma MDA levels in patients with prostate cancer in the study by Battisti et al. (38). However, they observed higher levels of carbonyl protein in patients with Gleason greater than 7 compared to patients with Gleason <7 and =7 (*p* < 0.05). Moreover, catalase activity, vitamin C, and vitamin E content were reduced in all groups in relation to the control group. Patients with advanced prostate cancer were subjected to high oxidative stress, as determined by the increased susceptibility of serum lipids to peroxidation (44). These results confirm that advanced prostate cancer is associated with a state of high oxidative stress.

In the final stage of our study, we examined whether the presence of perineural invasion impacts the levels of oxidative stress markers. Perineural invasion is a characteristic feature of prostatic carcinoma. In needle core biopsies, approximately 25% of cases exhibit perineural invasion. However, in a screening population characterized by smaller lower-stage tumors, perineural invasion is observed only in 11% of cases (45). The presence of perineural invasion significantly reduces serum and urinary TAC measured by FRAP and, surprisingly, increases urinary AOPP concentration. To our knowledge, this study is the first to examine the impact of perineural invasion on oxidative stress levels in patients with prostate cancer.

Prostate cancer tissue has been shown to produce particularly large amounts of hydrogen peroxide, a ROS that can damage biomolecules (46). Furthermore, the high activity of xanthine oxidase, which is a major source of ROS in the circulation, could be one of the causes of oxidative stress (27). In prostate cancer, changes in the activity of antioxidant enzymes (e.g., catalase, superoxide dismutase, glutathione peroxidase) and a decrease in the concentration of serum vitamins acting as antioxidants are also observed (38–40, 47). It should be noted that the type of prostate cancer treatment can also affect the level of oxidative stress (38, 48, 49). High levels of oxidative stress in prostate cancer are a complex phenomenon resulting from dysregulation of multiple metabolic and biochemical processes. Mitochondria are the main site of ROS production in cells, and their malfunctioning can result in excessive generation of these harmful molecules (50). A high level of ROS can lead to activation of ROS-dependent signaling pathways, such as the mitogen-activated protein kinase and nuclear factor kappa B pathways. These signaling pathways are involved in the regulation of cell proliferation, survival, migration, and invasion, which may contribute to the progression of prostate cancer. Moreover, activation of the nuclear factor kappa B pathway by ROS can lead to increased expression of genes promoting the survival of cancer cells, inflammatory cytokines, and growth factors, thus promoting the progression of prostate cancer (51). Understanding these mechanisms may be crucial for the development of targeted therapies that can inhibit disease progression by blocking ROS-dependent signaling pathways (52).

Oxidative stress can lead to gene mutations, including those in tumor suppressor genes that control cell growth and prevent uncontrolled cell division. DNA damage by ROS and RNS can promote the accumulation of mutations, a crucial step in cancer development (53). Moreover, oxidative stress can affect inflammatory processes, which are also linked to the development of prostate cancer. Inflammatory states can promote cancer cell growth and facilitate angiogenesis, essential for tumor nutrient supply (54). Laboratory studies suggest that antioxidants might help reduce oxidative stress and decrease the risk of developing prostate cancer. However, epidemiological studies on the impact of antioxidants on prostate cancer risk are inconclusive, and there is no definitive evidence of the effectiveness of antioxidants in prostate cancer prevention (55).

These results indicate a significant role of oxidative damage in prostate carcinogenesis. However, it is important to acknowledge the limitations of our study. We conducted our research on a small group of patients from a single clinic; thus, further research on a larger group of prostate cancer patients is necessary. We analyzed only selected oxidative stress parameters, and therefore, we cannot fully characterize the oxidoreductive balance in patients and controls. In the future, it would be beneficial to evaluate the relationship between oxidative stress intensity and the survival rate of prostate cancer patients. It is important to recognize that prostate cancer development is multifactorial, depending on various factors such as genetics, age, diet, environment, and lifestyle. Oxidative stress is just one of many factors that can influence the risk of this disease, and ongoing research is focused on understanding more specific mechanisms of this relationship.

## 5 Conclusions

In conclusion, our research indicates that increased oxidative stress occurs in patients with prostate cancer. As the tumor progressed and the presence of perineural invasion increased, oxidative stress increased. Assessing oxidative stress in these patients may aid in future prevention, diagnosis, and treatment. Additionally, characterizing oxidative and nitrosative damage to proteins may be useful in designing targeted therapies for prostate cancer patients.

## Data availability statement

The original contributions presented in the study are included in the article/supplementary material, Further inquiries can be directed to the corresponding author.

## Ethics statement

The studies involving humans were approved by Bioethics Committee of Rzeszow University. The studies were conducted in accordance with the local legislation and institutional requirements. The participants provided their written informed consent to participate in this study.

## Author contributions

MB: Conceptualization, Investigation, Software, Writing – original draft, Writing – review & editing. MM: Investigation, Methodology, Writing – original draft. KB: Resources, Supervision, Writing – original draft. ZK: Investigation, Writing – original draft. SG: Investigation, Methodology, Writing – review & editing.

## References

[1] Rawla P. Epidemiology of prostate cancer. World J Oncol. (2019) 10:63–89. doi: 10.14740/wjon1191 31068988 PMC6497009

[2] Osuchowski M Aebisher D Gustalik J Bartusik-Aebisher D Kaznowska E. The advancement of imaging in diagnosis of prostate cancer. Eur J Clin Exp Med. (2019) 17:67–70. doi: 10.15584/ejcem

[3] Zhang W Cao G Wu F Wang Y Liu Z Hu H. Global burden of prostate cancer and association with socioeconomic status, 1990–2019: A systematic analysis from the global burden of disease study. J Epidemiol Glob Health. (2023) 13:407–21. doi: 10.1007/s44197-023-00103-6 PMC10469111 37147513

[4] Udensi UK Tchounwou PB. Oxidative stress in prostate hyperplasia and carcinogenesis. J Exp Clin Cancer Res. (2016) 35:139. doi: 10.1186/s13046-016-0418-8 27609145 PMC5017015

[5] Shukla S Srivastava JK Shankar E Kanwal R Nawab A Sharma H. Oxidative stress and antioxidant status in high-risk prostate cancer subjects. Diagnostics. (2020) 10:126. doi: 10.3390/diagnostics10030126 32120827 PMC7151307

[6] Oczkowski M Dziendzikowska K Pasternak-Winiarska A Włodarek D Gromadzka-Ostrowska J. Dietary factors and prostate cancer development, progression, and reduction. Nutrients. (2021) 13:496. doi: 10.3390/nu13020496 33546190 PMC7913227

[7] Galiniak S Biesiadecki M Mołoń M Olech P Balawender K. Serum oxidative and nitrosative stress markers in clear cell renal cell carcinoma. Cancers (Basel). (2023) 15:3995. doi: 10.3390/cancers15153995 37568812 PMC10417121

[8] Galiniak S Mołoń M Biesiadecki M Mokrzyńska A Balawender K. Oxidative stress markers in urine and serum of patients with bladder cancer. Antioxidants (Basel). (2023) 12:277. doi: 10.3390/antiox12020277 36829836 PMC9952604

[9] Sawicka E Kratz EM Szymańska B Guzik A Wesołowski A Kowal P. Preliminary study on selected markers of oxidative stress, inflammation and angiogenesis in patients with bladder cancer. Pathol Oncol Res. (2020) 26:821–31. doi: 10.1007/s12253-019-00620-5 PMC7242270 30828780

[10] Schieber M Chandel NS. ROS function in redox signaling and oxidative stress. Curr Biol. (2014) 24:R453–62. doi: 10.1016/j.cub.2014.03.034 PMC4055301 24845678

[11] Aranda-Rivera AK Cruz-Gregorio A Arancibia-Hernández YL Hernández-Cruz EY Pedraza-Chaverri J. RONS and oxidative stress: an overview of basic concepts. Oxygen. (2022) 2:437–78. doi: 10.3390/oxygen2040030

[12] Tan BL Norhaizan ME. Oxidative stress, diet and prostate cancer. World J Mens Health. (2021) 39:195–207. doi: 10.5534/wjmh.200014 32648373 PMC7994655

[13] Gupta-Elera G Garrett AR Robison RA O’Neill KL. The role of oxidative stress in prostate cancer. Eur J Cancer Prev. (2012) 21:155–62. doi: 10.1097/CEJ.0b013e32834a8002 21857523

[14] Marhold M Kramer G Krainer M Le Magnen C. The prostate cancer landscape in Europe: current challenges, future opportunities. Cancer Lett. (2022) 526:304–10. doi: 10.1016/j.canlet.2021.11.033 34863887

[15] TNM classification of Malignant tumours. UICC. Available online at: https://www.uicc.org/resources/tnm-classification-malignant-tumours-8th-edition (Accessed 9 January 2024).

[16] Cooperberg MR Pasta DJ Elkin EP Litwin MS Latini DM Du Chane J. The University of California, San Francisco cancer of the prostate risk assessment score: A straightforward and reliable preoperative predictor of disease recurrence after radical prostatectomy. J Urol. (2005) 173:1938–42. doi: 10.1097/01.ju.0000158155.33890.e7 PMC2948569 15879786

[17] Lowry OH Rosebrough NJ Farr AL Randall RJ. Protein measurement with the folin phenol reagent. J Biol Chem. (1951) 193:265–75. doi: 10.1016/S0021-9258(19)52451-6 14907713

[18] Badiou S Dupuy AM Descomps B Cristolead JP. Comparison between the enzymatic vitros assay for creatinine determination and three other methods adapted on the olympus analyzer. J Clin Lab Anal. (2003) 17:235–40. doi: 10.1002/jcla.10103 PMC6807945 14614747

[19] Witko-Sarsat V Friedlander M Khoa TN Capeillère-Blandin C Nguyen AT Canteloup S. Advanced oxidation protein products as novel mediators of inflammation and monocyte activation in chronic renal failure1, 2. J Immunol. (1998) 161:2524–32. doi: 10.4049/jimmunol.161.5.2524 9725252

[20] Ellman GL. Tissue sulfhydryl groups. Arch Biochem Biophysics. (1959) 82:70–7. doi: 10.1016/0003-9861(59)90090-6 13650640

[21] Johnson RN Metcalf PA Baker JR. Fructosamine: A new approach to the estimation of serum glycosylprotein. An index of diabetic control. Clin Chim Acta. (1983) 127:87–95. doi: 10.1016/0009-8981(83)90078-5 6825313

[22] Mironova R Niwa T Handzhiyski Y Sredovska A Ivanov I. Evidence for non-enzymatic glycosylation of escherichia coli chromosomal DNA. Mol Microbiol. (2005) 55:1801–11. doi: 10.1111/j.1365-2958.2005.04504.x 15752201

[23] Re R Pellegrini N Proteggente A Pannala A Yang M Rice-Evans C. Antioxidant activity applying an improved ABTS radical cation decolorization assay. Free Radic Biol Med. (1999) 26:1231–7. doi: 10.1016/S0891-5849(98)00315-3 10381194

[24] Benzie IF Strain JJ. The ferric reducing ability of plasma (FRAP) as a measure of “Antioxidant power”: the FRAP assay. Anal Biochem. (1996) 239:70–6. doi: 10.1006/abio.1996.0292 8660627

[25] Yagi K. Assay for blood plasma or serum. Methods Enzymol. (1984) 105:328–31. doi: 10.1016/S0076-6879(84)05042-4 6727672

[26] Oh B Figtree G Costa D Eade T Hruby G Lim S. Oxidative stress in prostate cancer patients: A systematic review of case control studies. Prostate Int. (2016) 4:71–87. doi: 10.1016/j.prnil.2016.05.002 27689064 PMC5031904

[27] Veljković A Hadži-Dokić J Sokolović D Bašić D Veličković-Janković L Stojanović M. Xanthine oxidase/Dehydrogenase activity as a source of oxidative stress in prostate cancer tissue. Diagnostics (Basel). (2020) 10:668. doi: 10.3390/diagnostics10090668 32899343 PMC7555171

[28] Koike A Robles BEF da Silva Bonacini AG de Alcantara CC Reiche EMV Dichi I. Thiol groups as a biomarker for the diagnosis and prognosis of prostate cancer. Sci Rep. (2020) 10:9093. doi: 10.1038/s41598-020-65918-w 32499542 PMC7272452

[29] Goswami K Nandeesha H Koner BC Nandakumar DNA. Comparative study of serum protein-bound sialic acid in benign and Malignant prostatic growth: possible role of oxidative stress in sialic acid homeostasis. Prostate Cancer Prostatic Dis. (2007) 10:356–9. doi: 10.1038/sj.pcan.4500965 17404581

[30] Andrés CMC Pérez de la Lastra JM Andrés Juan C Plou FJ Pérez-Lebeña E. Impact of reactive species on amino acids-biological relevance in proteins and induced pathologies. Int J Mol Sci. (2022) 23:14049. doi: 10.3390/ijms232214049 36430532 PMC9692786

[31] Sajjaboontawee N Supasitthumrong T Tunvirachaisakul C Nantachai K Snabboon T Reiche EMV. Lower thiol, glutathione, and glutathione peroxidase levels in prostate cancer: A meta-analysis study. Aging Male. (2020) 23:1533–44. doi: 10.1080/13685538.2020.1858048 33325316

[32] Cimino S Favilla V Russo GI Galvano F Li Volti G Barbagallo I. Oxidative stress and body composition in prostate cancer and benign prostatic hyperplasia patients. Anticancer Res. (2014) 34:5051–6. 25202090

[33] Pizent A Anđelković M Tariba Lovaković B Živković Semren T buha djordjevic A gamulin M. Environmental exposure to metals, parameters of oxidative stress in blood and prostate cancer: results from two cohorts. Antioxidants (Basel). (2022) 11:2044. doi: 10.3390/antiox11102044 36290767 PMC9598453

[34] Yang S Pinney SM Mallick P Ho S-M Bracken B Wu T. Impact of oxidative stress biomarkers and carboxymethyllysine (an advanced glycation end product) on prostate cancer: A prospective study. Clin Genitourin Cancer. (2015) 13:e347–351. doi: 10.1016/j.clgc.2015.04.004 PMC4564335 25972296

[35] Twarda-Clapa A Olczak A Białkowska AM Koziołkiewicz M. Advanced glycation end-products (AGEs): formation, chemistry, classification, receptors, and diseases related to AGEs. Cells. (2022) 11:1312. doi: 10.3390/cells11081312 35455991 PMC9029922

[36] Chou Y-E Hsieh M-J Wang S-S Lin C-Y Chen Y-Y Ho Y-C. The impact of receptor of advanced glycation end-products polymorphisms on prostate cancer progression and clinicopathological characteristics. J Cell Mol Med. (2021) 25:10761–9. doi: 10.1111/jcmm.17025 PMC8581310 34708514

[37] Pande D Negi R Karki K Dwivedi US Khanna RS Khanna HD. Simultaneous progression of oxidative stress, angiogenesis, and cell proliferation in prostate carcinoma. Urol Oncol. (2013) 31:1561–6. doi: 10.1016/j.urolonc.2012.04.012 22591747

[38] Battisti V Maders LDK Bagatini MD Reetz LGB Chiesa J Battisti IE. Oxidative stress and antioxidant status in prostate cancer patients: relation to gleason score, treatment and bone metastasis. BioMed Pharmacother. (2011) 65:516–24. doi: 10.1016/j.biopha.2011.06.003 21993000

[39] Aydin A Arsova-Sarafinovska Z Sayal A Eken A Erdem O Erten K. Oxidative stress and antioxidant status in non-metastatic prostate cancer and benign prostatic hyperplasia. Clin Biochem. (2006) 39:176–9. doi: 10.1016/j.clinbiochem.2005.11.018 16413012

[40] Ahmed Amar SA Eryilmaz R Demir H Aykan S Demir C. Determination of oxidative stress levels and some antioxidant enzyme activities in prostate cancer. Aging Male. (2019) 22:198–206. doi: 10.1080/13685538.2018.1488955 30322333

[41] Perkovic MN Jaganjac M Milkovic L Horvat T Rojo D Zarkovic K. Relationship between 4-hydroxynonenal (4-HNE) as systemic biomarker of lipid peroxidation and metabolomic profiling of patients with prostate cancer. Biomolecules. (2023) 13:145. doi: 10.3390/biom13010145 36671530 PMC9855859

[42] Brys M Morel A Forma E Krzeslak A Wilkosz J Rozanski W. Relationship of urinary isoprostanes to prostate cancer occurence. Mol Cell Biochem. (2013) 372:149–53. doi: 10.1007/s11010-012-1455-z PMC3506833 22983829

[43] Zenner ML Helou YB Deaton RJ Sverdlov M Wang H Kajdacsy-Balla A. Advanced glycation end-products (AGEs) are lower in prostate tumor tissue and inversely related to proportion of West African ancestry. Prostate. (2022) 82:306–13. doi: 10.1002/pros.24273 PMC8753724 34855273

[44] Yossepowitch O Pinchuk I Gur U Neumann A Lichtenberg D Baniel J. Advanced but not localized prostate cancer is associated with increased oxidative stress. J Urol. (2007) 178:1238–1243; discussion 1243-1244. doi: 10.1016/j.juro.2007.05.145 17698111

[45] Bismar TA Lewis JS Vollmer RT Humphrey PA. Multiple measures of carcinoma extent versus perineural invasion in prostate needle biopsy tissue in prediction of pathologic stage in a screening population. Am J Surg Pathol. (2003) 27:432–40. doi: 10.1097/00000478-200304000-00002 12657927

[46] Moloney JN Cotter TG. ROS signalling in the biology of cancer. Semin Cell Dev Biol. (2018) 80:50–64. doi: 10.1016/j.semcdb.2017.05.023 28587975

[47] Kaba M Pirincci N Demir H Verep S. Serum prolidase activity, oxidative stress, and antioxidant enzyme levels in patients with prostate cancer. Urol Oncol. (2024) 42(4):116.e9-116.e15. doi: 10.1016/j.urolonc.2024.01.007 38341363

[48] Liu J Wang Z. Increased oxidative stress as a selective anticancer therapy. Oxid Med Cell Longev. (2015) 2015:294303. doi: 10.1155/2015/294303 26273420 PMC4529973

[49] Iynem AH Alademir AZ Obek C Kural AR Konukoğlu D Akçay T. The effect of prostate cancer and antiandrogenic therapy on lipid peroxidation and antioxidant systems. Int Urol Nephrol. (2004) 36:57–62. doi: 10.1023/b:urol.0000032676.31470.b2 15338676

[50] Han C Wang Z Xu Y Chen S Han Y Li L. Roles of reactive oxygen species in biological behaviors of prostate cancer. BioMed Res Int. (2020) 2020:1269624. doi: 10.1155/2020/1269624 33062666 PMC7538255

[51] Huang R Chen H Liang J Li Y Yang J Luo C. Dual role of reactive oxygen species and their application in cancer therapy. J Cancer. (2021) 12:5543–61. doi: 10.7150/jca.54699 PMC8364652 34405016

[52] Bahar ME Kim HJ Kim DR. Targeting the RAS/RAF/MAPK pathway for cancer therapy: from mechanism to clinical studies. Signal Transduct Target Ther. (2023) 8:455. doi: 10.1038/s41392-023-01705-z 38105263 PMC10725898

[53] Iqbal MJ Kabeer A Abbas Z Siddiqui HA Calina D Sharifi-Rad J. Interplay of oxidative stress, cellular communication and signaling pathways in cancer. Cell Communication Signaling. (2024) 22:7. doi: 10.1186/s12964-023-01398-5 38167159 PMC10763046

[54] Reuter S Gupta SC Chaturvedi MM Aggarwal BB. Oxidative stress, inflammation, and cancer: how are they linked? Free Radic Biol Med. (2010) 49:1603–16. doi: 10.1016/j.freeradbiomed.2010.09.006 PMC2990475 20840865

[55] Rago V Di Agostino S. Novel insights into the role of the antioxidants in prostate pathology. Antioxidants (Basel). (2023) 12:289. doi: 10.3390/antiox12020289 36829848 PMC9951863

