# Corrigendum: Shedding light on the shadows: oxidative stress and its pivotal role in prostate cancer progression

**DOI:** 10.3389/fonc.2024.1468582

**Published:** 2024-08-20

**Authors:** Marek Biesiadecki, Mateusz Mołoń, Krzysztof Balawender, Zofia Kobylińska, Sabina Galiniak

**Affiliations:** ^1^ Institute of Medical Sciences, Rzeszów University, Rzeszów, Poland; ^2^ Institute of Biology, Rzeszów University, Rzeszów, Poland

**Keywords:** oxidative stress, nitrosative stress, oxidative damage of protein, prostate cancer, cancer progression

In the published article, there was an error in **Tables 3**–**7** as published. The unit of serum Amadori products should be nmol/mg protein instead of μmol/mg protein. A typical unintentional editing mistake was made. The corrected **Tables 3**–**7** appear below.

**Table 3:** Markers of oxidative stress in healthy controls and patients with prostate cancer.

		Healthy controls	Prostate cancer group	p
Serum	AOPP (nmol/mg protein)	213.83 (192.02 − 291.16)	239.55 (195.37 − 425.47)	0.346
	3-nitrotyrosine (nmol/mg protein)	0.116 (0.11 − 0.14)	0.119 (0.1 − 0.16)	0.748
	Thiol groups (mmol/L)	643.92 (557.17 − 753.72)	573.87 (485.81 − 699.9)	0.044
	Amadori products (nmol/mg protein)	1745.69 (1587.43 − 2176.05)	1987.22 (1826.28 − 2299.01)	0.031
	TAC (ABTS^•^, μmol TE/L)	285.27 (271.48 − 294.88)	210.15 (201.85 − 217.55)	<0.001
	TAC (FRAP, μmol TE/L)	217.55 (184.24 − 237.92)	193.13 (168.65 − 225.12)	0.133
	MDA (μmol)	3.27 (3.11 − 3.41)	3.56 (3.39 − 4.02)	<0.001
	4-HNE (pg/mL)	345.62 (238.01 − 508.88)	412.08 (301.17 − 487.3)	0.746
Urine	AOPP (μmol/mmol creatinine)	12.72 (9.96 − 17.11)	14.87 (9.03 − 19.98)	0.538
	Amadori products (mmol/mmol creatinine)	0.352 (0.28 − 0.48)	0.384 (0.31 − 0.54)	0.467
	TAC (ABTS^•^, μmol TE/L)	856.83 (720.44 − 963.18)	758.85 (573.7 − 1026.94)	0.194
	TAC (FRAP, μmol TE/L)	564.90 (448.23 − 625.55)	451.44 (297.01 − 638.48)	0.047
	MDA (μmol/mmol creatinine)	0.86 (0.71 − 1.01)	1.15 (0.96 − 1.34)	<0.001

**Table 4:** Markers of oxidative stress in patients divided in groups considering the cancer stage depending on the TNM classification.

		T1	T2	T3	p
Serum	AOPP (nmol/mg protein)	238.35	219.40	378.77	0.123
	3-nitrotyrosine (nmol/mg protein)	0.137	0.117	0.113	0.941
	Thiol groups (mmol/L)	699.59	564.23	436.08	0.011
	Amadori products (nmol/mg protein)	1865.81	1996.79	2064.07	0.719
	TAC (ABTS^•^, μmol TE/L)	220.32	214.61	198.77	<0.001
	TAC (FRAP, μmol TE/L)	223.62	195.48	160.32	<0.001
	MDA (μmol)	3.39	3.56	4.31	0.001
	4-HNE (pg/mL)	246.9	417.42	508.19	0.002
Urine	AOPP (μmol/mmol creatinine)	12.21	17.83	15.37	0.594
	Amadori products (mmol/mmol creatinine)	0.384	0.357	0.382	0.405
	TAC (ABTS^•^, μmol TE/L)	763.28	696.62	959.94	0.589
	TAC (FRAP, μmol TE/L)	462	377.24	635.39	0.464
	MDA (μmol/mmol creatinine)	0.829	1.146	1.191	0.026

**Table 5:** Markers of oxidative stress in patients divided in groups considering Gleason grading system.

		<7	7	>7	p
Serum	AOPP (nmol/mg protein)	238.35	208.52	528.53	0.02
	3-nitrotyrosine (nmol/mg protein)	0.115	0.117	0.125	0.678
	Thiol groups (mmol/L)	700.81	555.68	488.69	0.002
	Amadori products (nmol/mg protein)	1829.38	2202.23	1962.49	0.086
	TAC (ABTS^•^, μmol TE/L)	215.74	215.24	198.77	<0.001
	TAC (FRAP, μmol TE/L)	207.85	216.93	166.00	0.007
	MDA (μmol)	3.40	3.42	4.01	0.077
	4-HNE (pg/mL)	258.68	417.42	527.98	<0.001
Urine	AOPP (μmol/mmol creatinine)	12.65	14.02	19.98	0.245
	Amadori products (mmol/mmol creatinine)	0.433	0.356	0.392	0.405
	TAC (ABTS^•^, μmol TE/L)	942.11	607.90	727.74	0.314
	TAC (FRAP, μmol TE/L)	634.48	446.00	498.74	0.70
	MDA (μmol/mmol creatinine)	0.853	1.174	1.306	0.054

**Table 6:** Markers of oxidative stress in patients divided in groups considering the cancer histological grade.

		G1	G2	p
Serum	AOPP (nmol/mg protein)	221.08	295.95	0.191
	3-nitrotyrosine (nmol/mg protein)	0.124	0.113	0.623
	Thiol groups (mmol/L)	623.49	555.31	0.37
	Amadori products (nmol/mg protein)	1982.92	2021.04	0.696
	TAC (ABTS^•^, μmol TE/L)	212.51	205.67	0.004
	TAC (FRAP, μmol TE/L)	220.27	166.01	<0.001
	MDA (μmol)	3.39	4.03	<0.001
	4-HNE (pg/mL)	390.8	474.68	0.124
Urine	AOPP (μmol/mmol creatinine)	14.43	19.97	0.999
	Amadori products (mmol/mmol creatinine)	0.321	0.382	0.049
	TAC (ABTS^•^, μmol TE/L)	728.12	912.93	0.731
	TAC (FRAP, μmol TE/L)	320.11	631.82	0.023
	MDA (μmol/mmol creatinine)	0.97	1.34	0.005

**Table 7:** Markers of oxidative stress in patients divided in groups considering the presence of perineural invasion.

		Absent of perineural invasion	Presence of perineural invasion	p
Serum	AOPP (nmol/mg protein)	219.36	277.59	0.346
	3-nitrotyrosine (nmol/mg protein)	0.114	0.134	0.603
	Thiol groups (mmol/L)	542.56	586.98	0.223
	Amadori products (nmol/mg protein)	1962.49	2092.05	0.19
	TAC (ABTS^•^, μmol TE/L)	215.63	208.95	0.154
	TAC (FRAP, μmol TE/L)	207.92	176.32	0.036
	MDA (μmol)	3.52	3.84	0.147
	4-HNE (pg/mL)	326.92	453.66	0.102
Urine	AOPP (μmol/mmol creatinine)	10.63	22.56	<0.001
	Amadori products (mmol/mmol creatinine)	0.356	0.388	0.89
	TAC (ABTS^•^, μmol TE/L)	998.71	694.79	0.063
	TAC (FRAP, μmol TE/L)	638.48	296.42	<0.001
	MDA (μmol/mmol creatinine)	1.11	1.19	0.452

The authors apologize for this error and state that this does not change the scientific conclusions of the article in any way. The original article has been updated.

